# Normative values of the retinal macular thickness in a middle eastern population

**DOI:** 10.1186/s12886-020-01391-3

**Published:** 2020-04-07

**Authors:** Mouna M. AlSaad, Amjad T. Shatarat, Saif Aldeen S. AlRyalat

**Affiliations:** 1 Department of Special Surgery, School of Medicine, The University of Jordan, Queen Rania AlAbdullah Street, Amman, 11942 Jordan; 2 Department of Anatomy and Histology, School of Medicine, The University of Jordan, Queen Rania Al-Abdullah Street, Amman, 11942 Jordan; 3 School of Medicine, the University of Jordan, Queen Rania Al-Abdullah Street, Amman, 11942 Jordan

## Abstract

**Background:**

Since the normative value of the retinal macular thickness is undocumented in the Middle East, the aim of this work is to assess the normative values of the macular thickness in healthy eyes in a Middle Eastern population and its relationship with age, sex, and laterality.

**Methods:**

One hundred sixteen individuals were randomly selected from volunteers visiting the Jordan University Hospital in Amman, Jordan. Measurements were obtained using the Fourier domain optical coherence tomography (OCT). Multivariate regression models were developed to obtain predicted normative values with adjustment to candidate variables. In addition, the effect of age, sex and laterality were evaluated.

**Results:**

The average central fovea macular thickness was 229.5 (±30.85) um. The quadratic value of the retinal macular thickness decreased from the superior value of 299.71 (±23.67) um (*P* = .001) to the inferior value of 296.46 (±28.85) um(P = .001) and a nasal figure of 93.63 (±26.86) um(P = .001). The temporal area has the thinnest value of 293.43 (±30.78) um (*P* = 0.001). Central thickness was higher in males with a mean variation of 11.67 um (95% CI, 2.41 to 20.93) (*p* = 0.003). The thickness was highest within 3 mm diameter from the center and decreased towards the periphery Eye sidedness didn’t contribute to variability of the macular thickness. Furthermore, we found a significant difference between age and central macular thickness (*p* = 0.001), as age was a positive predictor for macular thickness.

**Conclusion:**

Our set of predicted normative data may be used to interrupt measurement of the macular thickness in Middle Eastern population. The average fovea macular thickness among Jordanians is consistent with previously reported values. Normative values from additional Middle Eastern. Population are required to appraise our model.

Detection of abnormal s values of patients is conducted by using OCT. The patients obtained values are compared versus the normal values. Most of the patients used to measure the normal values are of white race. If racial difference exists, then this difference should be kept in mind for more accurate diagnosis of macular diseases [1, 2].

Optical Coherence Tomography (OCT) is a non-invasive imaging technique that measures internal structures of biological systems. Specifically, it is useful for high resolution reproducible in-vivo imaging of the retinal structure; this ocular technology is a useful tool to ophthalmologists. For instance, high resolutions in vivo retinal images are essential for diagnosis and follow up of patients with macular edema [1, 3, 4]. The outcome of OCT based imaging is constantly developing with further iterations on the technology. In fact, the latest iterations include Gabor-domain optical coherence microscopy which can be useful in assessment of the cornea [5, 6]. Based on this understanding, the aforementioned changes can be detected early by imaging the macula using an Ocular Coherence Tomography that facilitates both thicknesses and morphology detection before these changes are clinically apparent. In effect, early detection may favorably affect the visual outcome [7]. In the clinic, Fourier-domain OCT is used in standard commercial systems and offers superior sensitivity compared to the conventional time-domain approach [7, 8].

Retinal macular thickness is naturally subject to anatomic variation. Therefore, measurements are interpreted against a backdrop of normative reference values. Normative values are readily available, albeit for no more than a select number of ethnic groups [8]. The preceding fact is problematic as normative values may be highly variable between populations. Thus, their documentation in additional populations is necessary [8]. Therefore, detecting the normal value of the macular retinal thickness in a Middle Eastern population helps in early diagnosis of diabetic changes. Currently, data concerning the normal value of the adult Middle Eastern population, and the effect of age, sex, as well as the refractive error of the macular retinal thickness, is unavailable. As such, it is important to note that this aspect may be confused with early changes related to diabetic retinopathy [4]. The heterogeneity of Middle Eastern populations calls for a series of investigations to determine robust normative values of retinal macular thickness. Herein we present a preliminary investigation of these values using Fourier-domain OCT.

## Methods

**Fig. 1:**
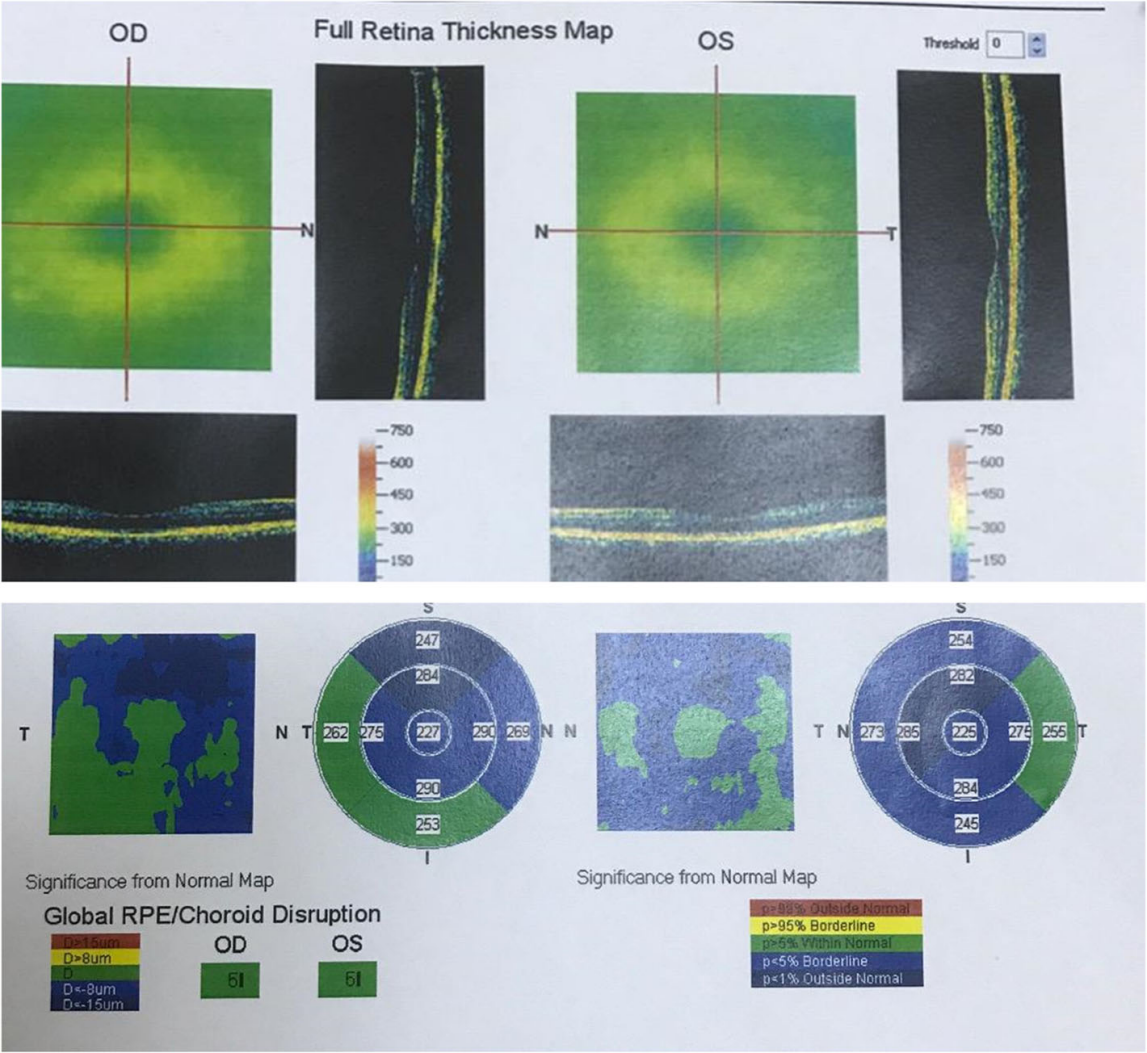
The 6 mm diameter macular retinal thickness map centered on the fovea to cover the 9 Early Treatment Diabetic Retinopathy Study (ETDRS) areas

For this retrospective cross-sectional study, patients who were evaluated in the ophthalmology clinic in the Jordan University Hospital were included. Data was collected from July 2017 to July 2018 after obtaining approval from our institutional review board. Written informed consent was obtained from all participants. The sampled subjects were adults aged above 18 years who underwent a complete ophthalmic assessment at the Jordan University hospital and whose data regarding macular OCT was available. The study’s exclusion criteria disqualified individuals with any history of ocular pathology, abnormal ocular exam comprising abnormally looking macula or diabetic retinopathy, recent history of trauma or ocular surgery (in the last 12 months), a high degree myopia (more than 6 diopters) or poor quality images. Retinal macular thickness was measured using Fournier domain OCT. A Macular cross line Emm5 protocol was used. These measurements were taken by the same operator in all cases. Measurements of the central fovea area and the macular scan were covered by an area of 6 mm2. The automated Optivue (RTVue, Optivue, Inc., Fremont, Canada) software derived a 6 mm diameter macular retinal thickness map centered on the fovea to cover the 9 Early Treatment Diabetic Retinopathy Study (ETDRS) areas (Fig. 1). The areas are located in three rings of 1, 3, and 6 mm diameters. The 1 mm ring covers the central fovea and the para-foveal area. In contrast, the other rings are located 3 and 6 mm from the 1 mm diameter ring. Each ring is divided into four quadrants that are described as superior, inferior, temporal, and nasal. Furthermore, age, sex, eye laterality and spherical equivalent were recorded. The IBM SPSS version 21.0 (Chicago, USA) is used in the present analysis. Similarly, the mean (± standard deviation) was applied in describing the continuous variables, which are age and measurements. Count (frequency) was used to describe other nominal variables that comprised the participants’ gender and eyes. On the other hand, numerical data was presented according to the recommendations of TJ Cole [9]. Furthermore, a sample T test was used to perform the analysis of the mean difference between measurements and each gender as well as laterality. The obtained data was presented in an average of 95% confidence intervals (CI). The correlations between central thickness and thickness at each quadrant at 3 mm and at 6 mm were studied using Pearson’s correlation. Moreover, Pearson’s correlation was used to study the correlation between age and each measurement. It should be noted that all underlying assumptions were met unless otherwise indicated. Lastly, a *p*-value of 0.05 was adopted as a significant threshold.

## Results

A total of 116 patients were included in this study, we included one eye per patient, with a mean age of 59.33 (±13.18) years. They were 45(38.3) men and 71 (61.7) women. The mean central macular thickness for the included sample was 229.5 (±30.85), (Table 1) present central macular thickness (1 mm ring) and thickness at 3 mm and 6 mm from the central ring.

**Table 1:** Central macular thickness (1 mm ring) and thicknesses at 3 and 6 mm from the central ring

	Total		Sex				Eye			
			Male		Female		Right		Left	
	Mean	SD	Mean	SD	Mean	SD	Mean	SD	Mean	SD
Central Thickness	229.50	30.67	234.76	36.30	225.88	26.18	231.98	32.20	226.50	28.91
Superior 3 mm	297.43	25.59	294.10	33.05	299.49	19.47	299.08	27.23	295.72	23.81
Nasal 3 mm	291.05	28.71	289.53	36.30	291.99	22.89	297.16	31.29	284.76	24.38
Inferior 3 mm	293.87	31.37	296.58	35.17	292.19	28.78	293.85	35.06	293.89	27.24
Temporal 3 mm	290.38	32.50	289.83	41.94	290.72	25.10	286.54	35.16	294.33	29.16
Superior 6 mm	280.58	29.14	279.62	29.46	281.18	29.03	281.24	30.14	279.91	28.20
Nasal 6 mm	282.73	33.62	284.48	33.30	281.64	33.90	293.28	36.00	271.86	27.12
Inferior 6 mm	273.64	31.48	274.19	32.89	273.30	30.70	272.19	34.39	275.14	28.26
Temporal 6 mm	284.92	30.04	287.19	32.96	283.51	28.12	275.76	29.87	294.34	27.30

Central macular thickness (1 mm ring) and thickness at 3 mm and 6 mm from the central ring

We grouped the age variable into < 30 years, 31–40 years, 41–50 years, 51–60 years, 61–70 years, and > 70 years. We used one-way ANOVA to analyze the difference in central macular thickness, with post-hoc Tukey test to find relation analysis. The median spherical equivalent was 0.5diopters (ranged from − 4.5 to 2.5 diopters). The participants comprised 45 men and 71 women. A total of 116 eyes were included in the present study, including 59 (50.8%) right side eyes, and 57 (49.2%) left side eyes. The mean central fovea macular thickness was 229.5 (±30.85) um (*p* = 0.001). The value of the Retina Macular thickness decreased from the superior value of 299.71 (±23.67) um (*p* = 0.001). to the inferior value of 296.46 (±28.85) um, (p = 0.001). to the nasal 293.63 (±26.86) um. (p = 0.001). The temporal has the thinnest value of 293.43 (±30.78) um. (p = 0.001). Table 1 presents the central macular thickness of 1 mm from the ring and thicknesses at 3 mm and 6 mm from the central ring.

**Fig. 2:**
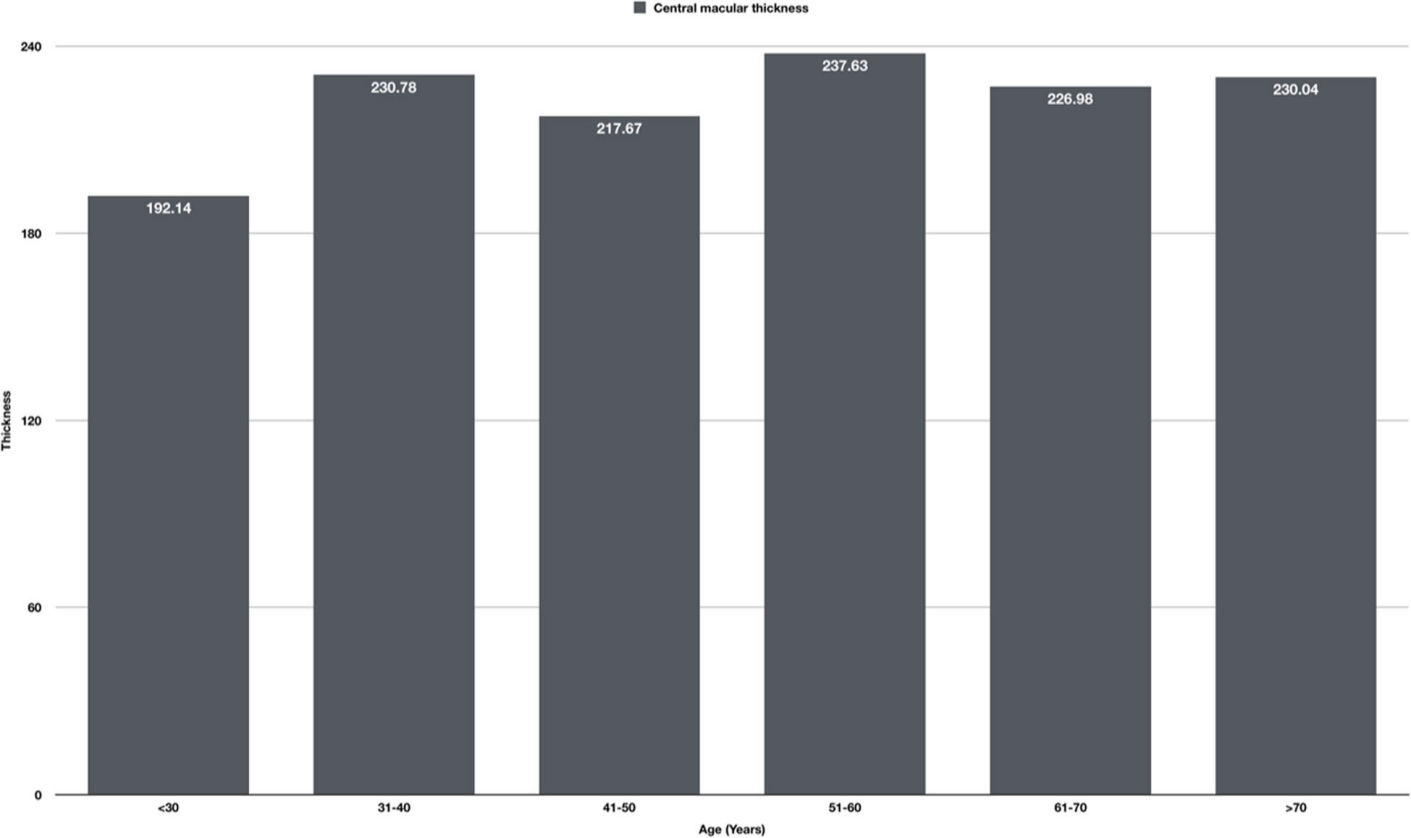
shows the central macular thickness according to the age group

We found a significant difference between age and central macular thickness (p = 0.001),the measurements gets thicker with age, with a post hoc test showing that the difference is between patients < 30 years and those between 51 and 60 years (*p* = 0.002), with a mean difference of 45.48 (95% CI: 11.92 to 79.05). Figure 2 shows the central macular thickness according to the age group.

We found significant gender differences for macular thickness at central thickness only (*p* = 0.042), higher in males with a mean difference of 8.88 (95% CI: 0.34 to 17.42). The central thickness for male was 234.76 (±36.30) and for female was 225.88 (±26.18). Upon comparing thickness differences between right and left eyes, we didn’t find any significant difference with any measurement.

Central thickness significantly and positively correlated with nasal at 3 mm (*p* < 0.001; correlation coefficient of 0.633), temporal at 3 mm (p < 0.001; correlation coefficient of 0.561), inferior at 3 mm (p < 0.001; correlation coefficient of 0.459), and superior at 3 mm (p < 0.001; correlation coefficient of 0.375).

The present study indicated significant gender differences for macular thickness at the following locations

- Central thickness (*p* = 0.003), higher in males with a mean difference of 11.67 um (95% CI: 2.41 to 20.93 um).
- Superior at 3 mm (*p* = 0.001), higher in females with a mean difference of 0.88 (95% CI: − 7.79 to 6.67 um).
- Nasal at 3 mm: (*p* = 0.002), higher in males with a mean difference of 0.56 (95% CI: − 5.67 to 10.71 um). (d) Temporal at 3 mm: (p = 0.001), higher in males with a mean difference of 0.30 (95% CI: − 4.41 to 14.33 um).

No gender differences were found for other measurements. Upon comparing thickness differences between right and left eyes, the present study did not find any significant difference with any performed measurement.

Central thickness significantly and positively correlated:

A) With nasal at 3 mm (*p* < 0.001; correlation coefficient of 0.60).
B) With temporal at 3 mm (p < 0.001; correlation coefficient of 0.52).
C) With inferior at 3 mm (p < 0.001; correlation coefficient of 0.38).
D) With superior at 3 mm (p < 0.001; correlation coefficient of 0.29).

The present study did not find a significant correlation between age and any investigated measurement. Refractive error didn’t statistically add to the prediction model. Predicted normative data are based on the regression model. The prediction assumes a negative history of systemic hypertension and a negative history of diabetes mellitus and a spherical equivalent of zero.

## Discussion

Optical Coherence Tomography is a new technique that can accurately measure the macular thickness in-vivo with high reproducibility [2]. Essentially, knowledge of the normal value of the macular thickness helps in early detection of any abnormalities [7]. We developed regression models to predict the normative values of retinal macular thickness in a Middle Eastern population. During model development, we examined retinal macular thickness measurements for sexual dimorphism, binocular asymmetry, age-related changes, and clinical association with refractive error. Ethnic variability, the effect of age, sex, distance from the fovea and laterality has been reported by various studies [8, 10–17]. Furthermore, the current work is consistent with the findings of most previous investigations. Based on a study conducted on the Caucasian population [8] the central fovea’s macular thickness was 278.2um (range 266-291um) using spectral domain OCT on Caucasian population. This inquiry is consistent with the present findings. Furthermore, male participants have a higher macular thickness in all areas except for the temporal superior and the outer segment after adjusting the age. In the present study, the mean value of the central fovea area was obtained as 232.1 (±30.85) um. This value is consistent with that of the previous studies on the Caucasian population using the Spectrally OCT 270+/− 22.5 um [4, 10]. Based on previous studies we have noticed that middle Eastern population sample investigated in our study had thinner central foveal thickness when compared to the Iranian population but thicker than other populations like African Americans, Japanese White American, Indians and blacks(5,11,16–20). Moreover, the results are variable when compared to other Caucasians [8]. Table 2 summarizes the central macular thickness and population.

**Table 2:** summarizes the central macular thickness and the ethnicity

Population	The central foveal Thickness
Caucasians population using spectral domain OCT	278.2 +/− 12 um (*P* = .038)
Iranian Population	255.4 um (P=,0.001)
African American	181.1+/_ 3.7 um (P = 0.001)
Japanese	209.5+/− 26.7um (*P* = 0.001)
USA	mean fovea 212+/− 20 um (*p* = 0.01)
Indians	149.19 + \- 21.15um (p = 0.01)
Blacks	160+/− 26 um (P < 0.001)

Various studies have evaluated the demographic variations in macular thickness [13–18]. A value of 181.1+/_ 3.7 as the normal figure for mean foveal thickness in African Americans and 200.27+/−27um was suggested in whites [15]. Additionally, it was concluded that blacks tend to have thinner retinas compared to whites using Stratus OCTs [16]. In addition, a significant difference in mean foveal thickness between Blacks and whites using Spectralis SD-OCT was documented. Moreover, a thinner mean foveal thickness was observed in healthy Indians compared to other populations that were found to have a value of 149.19 + \- 21.15um using Stratus OCT [18]. In contrast, it was observed that Japanese have thicker retinas compared to the US population using Stratus OCT [17]. Concerning the distribution of macular thickness in Iranian population, results show that the central foveal was 255.4 um, while the average inner thickness was 316.5 um. The average outer thickness was found to be 275.3 um, whereas the overall thickness was 278.6 um. All the obtained results from this study indicated a thicker central foveal in males compared to women. The central area of the fovea increased with age while the thickness in the other areas decreased. Other areas of the retina were evaluated in the Middle Eastern population namely, the peri-papillary nerve fiber layer, this analysis concluded that the thickness in various areas is consistent with previous studies [19], Essentially, the African-American race was a predictor of decreased mean foveal thickness when compared to Caucasians and Hispanics.

This investigative inquiry reveals that age is positively correlated with macular thickness. This result is inconsistent with a previous work [11], which demonstrated that age had a negative correlation with all ETRDS macular areas except those found in the central fovea areas. The retinal thickness values of the present inquiry were thinnest in the fovea area and thickest in the para-foveal area. These values decrease as the distance from the fovea increases. This finding is consistent with previous investigations [12], whose analysis concluded that irrespective of age or sex, macular thickness increased when moving from the central fovea area to the 3 mm area. Eventually, it was thinnest in the 6 mm area. However, in that study, the central thickness was 245.44+/− 20.39um, which is thicker compared to the results obtained in this investigation. Concerning eye sidedness, previous investigative inquiries indicated that the average macular thickness had no significant difference between the right and the left eye [13]. This finding is consistent with the results of the current study. Furthermore, males were associated with increased mean foveal thickness. Similarly, a negative correlation between age and thickness was reported [4], while other investigators concluded that there is no effect of age and gender on macular thicknesses appears, then, that adjustments need not be made to the retinal macular thickness measurements in Middle Easterners. However, the Middle Eastern population comprises a heterogeneous admixture of peoples. Therefore, normative data from additional Middle Eastern populations are required to confirm this finding. Limitations of the study is that the sample is a clinic sample, although that the clinic is a tertiary referral center in which patients come from different areas to visit the hospital, this sample may not accurately represent the middle eastern population and further, studies from different areas and higher numbers size will help confirming our observation.

### Conclusion

The results of the present research indicate that in the Jordanian population (a Middle Eastern population) it is possible to interrupt the macular thickness more accurately.

Furthermore, our study concluded that the central foveal and macular thickness in the Middle Eastern population is different from many other ethnicities. Ethnicity based variations are important when assessing disease that may affect the macula. This should be taken in consideration when evaluating patients from different origins and reading the central macular thickness. in addition, machine manufactures should take in consideration the ethnicity of the population the machine is going to be used on in order to modify the normal values set.

This inquiry’s findings suggest that males have higher values. However, normative data from other Middle Eastern populations are required to appraise the models employed in this experiment.

## Data Availability

The data that support the findings of this study are available from the corresponding author upon reasonable request.

## Abbreviations

CI: Confidence interval
ETDRS: Early treatment diabetic retinopathy study
Mm: Millimeter
OCT: Ocular coherence tomography
Um: Micrometer
